# The new reconstruction technique in the treatment of the skin cancers located on the eyelid: Posterior temporalis fascia composite graft

**DOI:** 10.1186/1477-7800-1-5

**Published:** 2004-08-11

**Authors:** Eray Copcu, Nazan Sivrioglu

**Affiliations:** 1 Plastic and Reconstructive Surgery Department, Medical Faculty, Adnan Menderes University, 09100, Aydin, TURKEY

## Abstract

**Background:**

Difficulty of reconstruction of the eyelids arises from the need to reconstruct different supporting and covering structures in a single operation. Defects in the anterior lamella of the eyelids can be readily repaired with skin grafts or flaps but posterior lamellar reconstruction needs more complex applications.

**Methods:**

We performed posterior lamellar eyelid reconstruction with posterior parts of the temporalis fascia, since their anatomical and histological features are very similar to the defects. Nine patients with skin tumors located on the periorbital region were treated with local skin flaps and deep layer of the temporalis fascia.

**Results:**

Grafts were harvested very easily. There was no complication related with graft or donor site. Biopsy was performed in three cases and normal conjunctival elements were seen. Functional and acceptable aesthetically results were achieved in all patients.

**Conclusion:**

Ideal reconstructive material for replacement of the posterior lamina is still lacking. Tarsal reconstruction can be made with deep temporalis fascia with success since the thickness of the both tissues are very similar and also since the loose areolar layer of the temporalis fascia is very thin and highly vascularized, this layer can be used in reconstruction of the conjunctiva. According to our knowledge this is the first report of using of the posterior part of temporalis fascia as a composite graft in the literature.

## Introduction

Reconstruction of eyelid defects after tumor excision should aim at obtaining full globe protection without visual disruption and restoring the area to an appearance as close to normal as possible [1]. Reconstruction of the eyelids requires special considerations and complete understanding of the specialized anatomy of the region. The eyelids consist of an anterior lamella of skin, orbicularis muscle; posterior lamella of tarsus and conjunctiva. Full thickness defects of eyelid after tumor resection require reconstruction of these layers. However reconstruction of the skin and subcutaneous tissue can be easily reconstructed with skin grafts and local flaps, most important subject is reconstruction of the posterior lamellar segment of the eyelid. Tarsal plate is dense, fibrous tissue (not a cartilage!) that gives the eyelid its contour and provides its skeleton [2]. Tarsal substitutes including banked sclera, nasal cartilage, ear cartilage, and periosteum can be beneficial for posterior lamellar repair [3]. Reconstruction of the conjunctiva is more complex issue.

Tarso-conjunctival grafts, buccal mucosa, hard palate mucosa and amniotic membrane have been used in the reconstruction of the conjunctival defects and all techniques were reported with individual advantages and disadvantages. Temporalis fascia has a specific and complex anatomy [4-6]. Mainly temporalis fascia consists of two layers: superficial temporalis fascia and deep temporalis fascia. The superficial temporalis fascia is separated from the deep temporalis fascia by a distinct plane of loose areolar tissue: loose areolar layer [5]. In our study we performed posterior lamellar reconstruction of the lower eyelid defects with loose areolar layer for conjunctiva and deep temporalis fascia for tarsus in the patients who required total eyelid reconstruction secondary to the skin tumors. Loose areolar layer (subaponeurotic layer or subgaleal fascia) has highly vascularized histological structures and this feature may allow growing of the bulbar conjunctiva very easily [4]. Since the deep layer of temporalis fascia is very similar to tarsus, reconstruction with deep layer temporalis fascia provides good structural stability as original skeleton. The cosmetic and functional outcomes of our technique are encouraging.

## Methods

Nine patients with lower eyelid defects after removal of malignant tumors were treated with local flaps combined with temporalis fascia grafting in the Department of the Plastic and Reconstructive Surgery, Adnan Menderes University Hospital. Data of the patients were summarized in Table 1.

**Table 1:** Clinical cases

No:	Age	Sex	Lesion	Size of defect (mm)	Flap type	Pathology	Complication
1	52	Female	Left lower eyelid	20 × 5	Mustardé	Basal cell Ca	None
2	66	Male	Left lower eyelid	38 × 12	Mustardé	Basal cell Ca	None
3	60	Male	Right lower eyelid	28 × 8	Mustardé	Basal cell Ca	None
4	48	Male	Right lower eyelid	26 × 10	Mustardé	Basal cell Ca	None
5	36	Female	Inner right canthus	40 × 10	Tri-lobed flap	Basal cell Ca	None
6	56	Female	Left lower eyelid	22 × 8	Bi-lobed flap	Basal cell Ca	None
7	71	Female	Right lower eyelid	45 × 10	Tri-lobed flap	Basal cell Ca	None
8	52	Male	Left inner canthus	42 × 15	Forehead flap	Basal cell Ca	None
9	21	Female	Right inner canthus	50 × 15	Forehead flap	Basal cell Ca	None

Ages of patients were ranged from 21 to 71. Full thickness defects measured from 15 × 5 mm to 50 × 15 mm. All patients were operated under the general anesthesia. The follow up periods ranged from 6 months to 24 months. After ethical approval from our department and patient consent, biopsy performed in three cases to evaluate the technique.

### Surgical Technique

**Figure 1:**
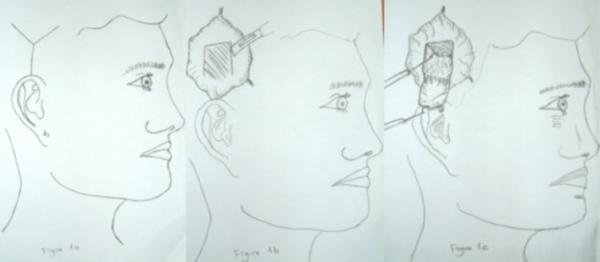
Schematic view of the graft harvesting. 1a skin incision, 1b: fascia incision 1c: harvesting of the fascia.

Tumor excision was performed with safety surgical margins in all patients. Mustardé cheek flap was prepared in four patients, forehead flap and tri-lobed flap in two patients and one bi-lobed flap were designed. "Y" skin incision was performed for harvesting of the temporalis fascia (Figure 1a,1b and 1c).

**Figure 2:**
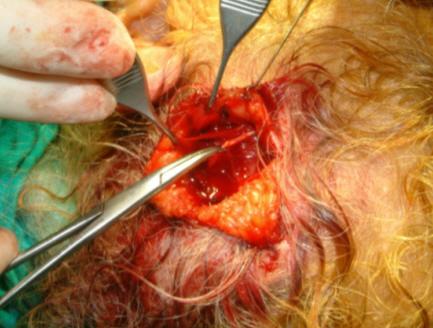
Three layers of the fascia of temporalis region. From above to below: superficial temporalis fascia, loose areolar fascia and deep layer. Loose areolar layer and deep layer can be harvested easily.

Against possible hair damage we did not use solution containing of epinephrine for haemostasis. The scalp is incised at angle that follows the direction of the hair follicles, which is facilitated by not shaving the hair. The incision is made through the scalp to the superficial fascia. Superficial fascia was incised to the temporalis muscle in dimensions 40 × 20 mm. This layer was elevated above the loose areolar tissue as distal based flap and loose areolar layer and deep temporalis fascia was dissected and harvested (Figure 2).

Superficial fascia was re-located in its original location. Fascia was sutured with 4-0 vicryl. Haemostasis was performed with bipolar cautery. Scalp was closed with 3-0 nylon sutures. Harvested fascia consisted of the layers: loose areolar tissue and deep temporalis fascia. Continuity of these two layers were not disrupted and used as a composite graft. Thin and membranous loose areolar tissue was used for conjunctiva reconstruction and dense and firm deep temporalis fascia was used for tarsus reconstruction. These two layers were adapted to the flap with 6-0 catgut sutures. Flaps were transposed to the defects. Loose areolar tissue was sutured to the edge of palpebral conjunctiva with continuous 7-0 vicryl. One edge of the deep temporalis muscle was sutured to the periosteum, which is located inside the lateral orbital rim and the other edge sutured to the medial canthal tendon with 4-0 nylon.

Prophylactic antibiotics were used topically in post-operative first week.

## Results

**Figure 3:**
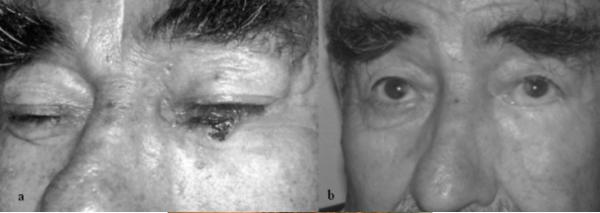
View of the patient. 3a: Patient with skin tumor located on lower eyelid. Tumor excision and Mustardé cheek flap with posterior temporal fascia graft were applied to this patient. 3b: Post-operative six months view of the reconstructed conjunctiva with loose areolar layer.

**Figure 4:**
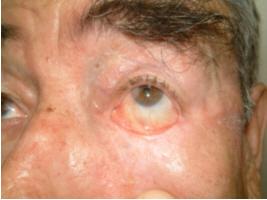
Post-operative six months of the patient. Conjunctiva was reconstructed with "loose areolar layer" of the temporalis fascia; tarsal plate was reconstructed with deep temporalis fascia. (Patient pulled down his eyelid with his finger)

**Figure 5:**
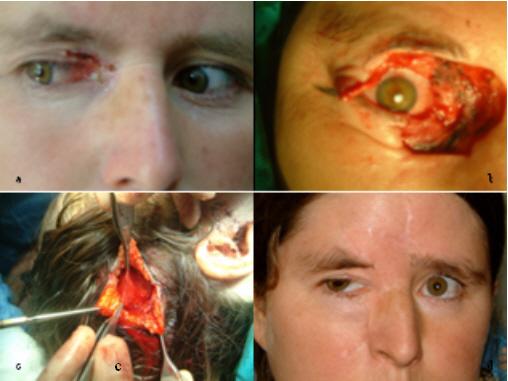
Figure View of the patient. 5a: Patient with skin tumor located on inner canthus and lower eyelid. Tumor excision and bi-lobed forehead skin flap with posterior temporal fascia graft were applied to this patient. 5b: View after excision of the tumor. 5c: Harvesting of the fascia for posterior lamella reconstruction. 5d: Post-operative six months view of the patient. There was no complication. Patient did not accept the further revision.

All patients tolerated the operation well (figure 3,4,5)

**Figure 6:**
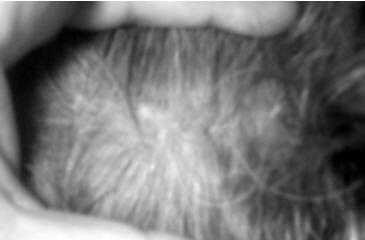
Post-operative six months view of the donor site. There is no scar.

There was no early or late complication in donor site and flap (Figure 6).

**Figure 7:**
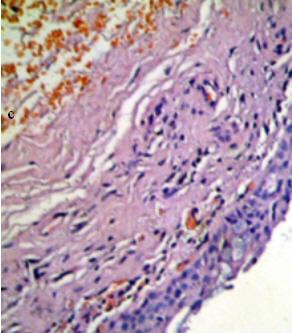
Biopsy form grafted area of the lower eyelid. Hematoxylene-eosin staining, × 100 magnification.

We observed neither infection nor irritation signs nor symptoms. There was no graft lysis. Patients were followed minimum six months. There were no complaints in this period in the patients. In all patients, the functional and aesthetic results were achieved. There were no signs and symptoms related with shrinkage of the grafts. Microscopically normal conjunctival elements were seen in the biopsy of the reconstructed conjunctiva with Hematoxylene-eosin staining (Figure 7).

## Discussion

Difficulty of the reconstruction of eyelids arises from the need to reconstruct different supporting and covering structures, i.e. the conjunctiva, tarsus, orbicularis muscle, canthal ligaments and skin. Many flaps and reconstruction techniques were described in the literature for anterior lamellar part of the eyelids. In our study, we used the posterior part of the temporalis fascia for the posterior lamellar part of the eyelids. Fox and Edgerton first used the fascia of the temporalis region in reconstructive surgery [7]. Brent and Byrd used the temporoparietal fascia for ear reconstruction and they also stated that these anatomical structures could be used as a free microvascular autograft [8]. Temporalis fascia widely used ophthalmic reconstructive material in plastic surgery especially in socket reconstruction as a flap [9]. This tissue were also used in open rhinoplasty, facial paralysis, Peyronie disease, reconstruction of temporomandibular joint, repair of the perforation of nasal septum, lip augmentation and finally malar augmentation were reported in the literature [10-16]. Superficial layer of the fascia was mainly used in all these studies. But temporalis region has a specific fascial anatomy. In the temporoparietal region, there are four and in some places five different layers, excluding the skin, subcutaneous tissue and the temporalis muscle [6]. Although many reports were presented in the literature about the anatomy of the fascia of temporoparietal region, there is no consensus in terminology. There are several names in current use for each layer. In our study we used posterior part of the tissue: subaponeurotic plane and deep temporal fascia.

The superficial temporal fascia is separated from the deep temporal fascia by distinct plane of loose areolar tissue [5]. This layer has been termed the "loose areolar layer" or "subaponeurotic layer" or "subgalael fascia". This tissue is well developed and highly rich supplied by the branches from the superficial temporal artery. It is easy to dissect as a discrete layer^6^. However we performed our operation under the general anesthesia, Miller pointed that the temporalis area can be easily anesthetized by infiltration local anesthesia [17]. The graft can be obtained quickly under the direct vision. There is minimal amount of postoperative pain and no visible scar. We speculate that highly vascularized histological structure of the loose areolar tissue allows growing of the bulbar conjunctiva very easily and conjunctival elements were seen in histological examinations in the biopsy from the graft in post-operative six months. Tolhurst et al. presented detailed anatomical research about the subgaleal fascia [6]. They summarized the advantages of this layer as:

(a) this layer is thin and will conform to the shape of underlying soft tissue or cartilage with aesthetically pleasing fidelity,

(b) well vascularized, and if handled with care, will readily support split and full thickness skin graft,

(c) can be harvested easily, with minimal donor-site morbidity.

We used this anatomical structure for conjunctiva reconstruction. Conjunctiva is a mucous membrane that covers the posterior aspect of the eyelids (palpebral conjunctiva) and the anterior surface of the globe (bulbar conjunctiva). Defects larger than 25% of the eyelid usually require a free graft to make for the loss of specialized tissue. Conjunctiva harvested from another lid is the ideal match and physiologically but it is thin, difficult the handle, has tendency to contract, and can only be harvested sparingly to avoid interfering to donor fornices. In addition this technique requires two operations when it is performed as a lid sharing procedure. Oral mucosa is abundant and simple to remove but tends to contract and infection. Nasal mucosa is easier to handle, and minimal contraction but this procedure has some problem of poor access and thickness. Today, most performed techniques are hard palate mucosal graft, amnion membrane, chondormucosal Septum [18-20]. Tarsal plate was reconstructed with deep layer of the temporalis fascia in our study. The deep temporalis fascia is a white, dense, though, uniform fascial layer similar in strength, appearance, and thickness to the sheet of the rectus abdominis muscle [5]. The tarsal plates are composed of dense fibrous connective tissue that provides structural support to the eyelid [2]. Matsumoto et al used the fascia lata for the reconstruction of the tarsal plate [21]. They performed this operation with cheek flap for skin coverage, and buccal mucosa graft for the conjunctiva defect. They applied two different graft materials (mucosa and fascia) to the flap.

One of the most popular grafts in posterior lamella reconstruction used to come from the chondromucosal nasal septum. Major disadvantages of this technique are the difficulty of the shape and adapt to the eyelid. Palatal mucosal grafts were widely used. Such grafts meet both the mucosal and supporting requirements. The use of hard palate mucosal grafting in lower eyelid reconstruction was first described by Siegel [22]. Hard palate is ideal material for posterior lamellar reconstruction but this technique has serious complications such as: infection, oronasal fistula, and post-operative discomfort. Also increased operating time for graft harvest and occasional keratinization of the surface with potential ocular surface irritation could be listed as disadvantages of the technique [23].

In our series we did not observe irritation signs and symptoms.

It is agreed among most authors that the ideal reconstruction should use identical or similar tissue to that of the original structure. Undoubtedly, reconstructions of the posterior lamella with original tissue i.e. free tarso-conjunctival grafts or flap is the best treatment modality. If the donor sites of the tarso-conjunctival grafts or flap were not suitable or enough, deep temporalis fascia grafting would be an alternative in the reconstruction of the posterior lamella.

We believe that application of the deeper part of the temporalis fascia for the reconstruction of the posterior lamella has many advantages: easy to perform, single composite graft for two layers, without or minimal donor site morbidity, no infection risk, no shrinkage of the graft, easy to adapt, wide donor site, no irritation and excellent cosmetically results. There is no data about the using of the posterior part of the temporalis fascia in reconstruction of the tarsus and conjunctiva in the literature.

In conclusion, loose layer of the temporalis fascia is ideal tissue for the growing of the conjunctiva and the thickness of the deep layer of the temporalis fascia is very similar to the tarsus. Both tissues can be used in reconstruction of the posterior lamellar reconstruction of the eyelids with success.

## Competing interests

None declared

## Authors' contributions

EC conceived of the study, and participated in its design and coordination. NS participated in design of study. All authors read and approved the final manuscript.
